# A Key Molecular Regulator, RNA G-Quadruplex and Its Function in Plants

**DOI:** 10.3389/fpls.2022.926953

**Published:** 2022-06-15

**Authors:** Haifeng Liu, Zhaohui Chu, Xiaofei Yang

**Affiliations:** ^1^State Key Laboratory of Crop Biology, College of Agronomy, Shandong Agricultural University, Taian, China; ^2^Department of Cell and Developmental Biology, John Innes Centre, Norwich Research Park, Norwich, United Kingdom; ^3^State Key Laboratory of Hybrid Rice, Hubei Hongshan Laboratory, College of Life Sciences, Wuhan University, Wuhan, China; ^4^National Key Laboratory of Plant Molecular Genetics, CAS Centre for Excellence in Molecular Plant Sciences, Shanghai, China; ^5^CAS-JIC Centre of Excellence for Plant and Microbial Science, Shanghai, China

**Keywords:** RNA structure, RNA G-quadruplex structure, gene expression, plant growth and development, evolution

## Abstract

RNA structure plays key roles in plant growth, development, and adaptation. One of the complex RNA structures is the RNA G-quadruplex (RG4) where guanine-rich sequences are folded into two or more layers of G-quartets. Previous computational predictions of RG4 revealed that it is widespread across the whole transcriptomes in many plant species, raising the hypothesis that RG4 is likely to be an important regulatory motif in plants. Recently, with the advances in both high-throughput sequencing and cell imaging technologies, RG4 can be detected in living cells as well as at the genome-wide scale. Here, we provide a comprehensive review of recent developments in new methods for detecting RG4 in plants. We also summarize the new functions of RG4 in regulating plant growth and development. We then discuss the possible role of RG4 in adapting to environmental conditions along with evolutionary perspectives.

## Introduction

A fundamental question of plant science is to decode the mechanisms of gene regulation that determine plant phenotypes. As a key molecule in the flux of gene expression, RNA not only carries the genetic blueprint for protein translation but also plays a fundamental role in regulating gene expression (Morris and Mattick, 2014). Accumulating evidence has shown that RNA structure dictates gene regulation at many post-transcriptional levels (Klaff et al., 1996; Wan et al., 2011; Yang X. et al., 2018). Through base-pairing between the nucleotides, RNA molecules can form into diverse structures, including both the canonical RNA structure elements, such as stem-loop and noncanonical RNA structure elements, such as RNA Guanine (G)-quadruplex (RG4) (Taylor and Sobczak, 2020). For decades, much effort has been attracted to studying the canonical RNA structures, while less is known about noncanonical RNA structures.

**Figure 1:**
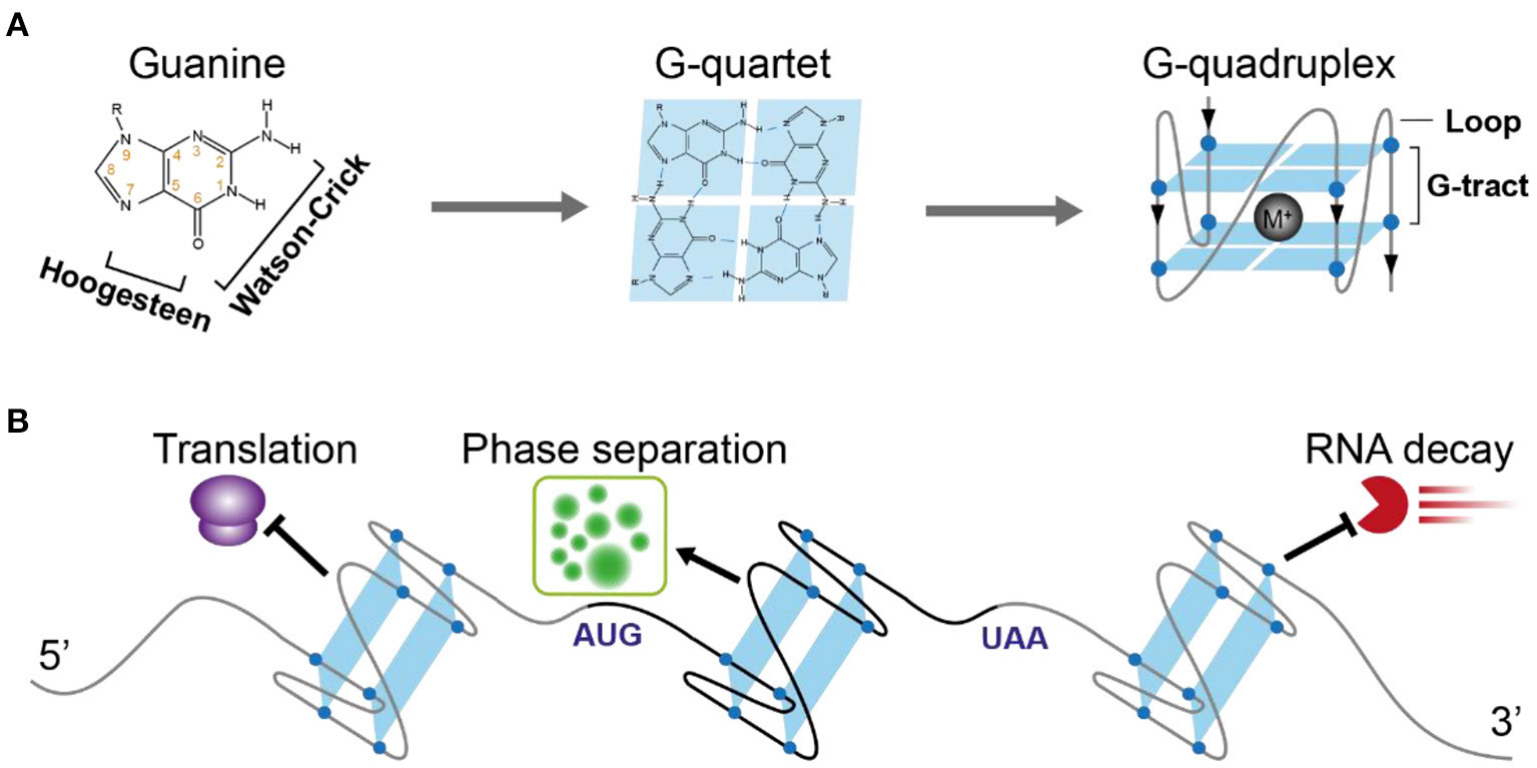
Schematic illustration of RNA G-quadruplex structure and its molecular function in plants. **(A)** The schematic depicts the guanine, G-quartet, and G-quadruplex respectively. The chemical structure illustrates the hydrogen bonds of Watson-Crick face and Hoogesteen face of guanine forming G-quartet. G-quadruplex with two layers of G-quartet was further illustrated, with a monovalent cation between the G-quartets stabilizing the G-quadruplex. **(B)** The versatile post-transcriptional regulatory functions of RNA G-quadruplex in plants. 5'UTR-located RNA G-quadruplex (*ATR, SMXL4/5, HIRD11*) represses translation, CDS-located RNA G-quadruplex (*SHR*) triggers phase separation, 3'UTR-located RNA G-quadruplex (*CORG1*) represses mRNA decay.

One of the famous noncanonical RNA structures is RG4, which is formed by a G-rich motif and consists of two or more layers of G-quartets involving both Hoogsteen and Watson-Crick base pairs (Figure 1A) (Varshney et al., 2020). The RG4 structure shows strong thermostability *in vitro* (Kwok et al., 2015; Kharel et al., 2020), proposing the high possibility that RG4 is folded *in vivo*. In mammalian cells and yeast, the *in vivo* folding of RG4 was originally denied (Guo and Bartel, 2016). However, *in vivo* folding had been recently confirmed in *Arabidopsis* and rice (Yang et al., 2020). Up to date, only a few cases have shown a potential link between RG4 structure and plant development (Cho et al., 2018; Yang et al., 2020). Since hundreds of RG4s are found in both dicots and monocots (Yang et al., 2020), they may have a broad functionality in plants.

The goal of this review is to summarize the knowledge of the intrinsic and extrinsic features affecting RG4 folding, the methods of prediction and detection of RG4 structure, the molecular functions of RG4 in gene regulation, and their biological significance, and to propose possible studies in future.

## Intrinsic and Extrinsic Features Affecting RNA G-Quadruplex Folding

A typical G-rich motif competent to fold into RNA G-quadruplex consists of four clusters of G-tract and loops separating the G-tracts (Figure 1A). The Watson-Crick and Hoogsteen base-pairing between four G-nucleotides enables the formation of the G-quartet (Figure 1A). Stacking of more than two layers of G-quartet drives the folding of RNA G-quadruplex, with the loops connecting G-tracts outside the stacked G-quartets. Both the number of G-quartet layers and loop length have a profound impact on the thermostability of RG4 (Pandey et al., 2013; Kwok and Merrick, 2017; Jana and Weisz, 2021). The most common RG4s are constituted with two layers of G-quartet (G2-RG4) or three layers of G-quartet (G3-RG4). In general, an increase of the G-quartet layers is likely to stabilize the RG4 structure, leading to higher stability of G3-RG4 over G2-RG4. In contrast to a strong enrichment of G3-RG4 in human cells (Kwok et al., 2016a), G2-RG4 is preferably enriched in *Arabidopsis* transcriptome (Mullen et al., 2010; Yang et al., 2020), suggesting a species-dependent selection on the RG4 structures. Besides the G-quartet layer, loop length provides another important layer of effect contributing to RG4 stability, an increase in the loop length has a negative impact on RG4 stability. In plants, there are more RG4s with longer loops than that with shorter loops (Garg et al., 2016; Yang et al., 2020).

RG4 formation is not only affected by the sequences inside the G-rich motif itself, but also by the sequences flanking the motif. The folding possibility of RG4 can be strongly declined by the presence of high C content inside and outside the motif, which could be explained by the competition of G-C Watson-Crick base-pairing over G-G base-pairing (Beaudoin et al., 2013; Kwok et al., 2016a). Different combination of sequences inside and outside the G-motif provides various patterns of sequence selection constraining RG4 folding possibility in a given genetic context.

*In vitro* folding of RG4s is strongly dependent on the conditions, such as temperature and cations. RG4 folding is stabilized by lower temperatures but destabilized by higher temperatures (Pandey et al., 2013; Kwok et al., 2015). RG4 formation could be promoted by the coordination of larger cations such as K^+^ rather than the smaller cations such as Li^+^, with their stabilizing effect in the following order: K^+^ > Na^+^ >> Li^+^ (Kwok et al., 2015; Kharel et al., 2020). Cations are not necessarily required for RG4 folding *in vitro*, in the absence of K^+^, the presence of RNA binding proteins (RBP) JULGI can promote RG4 formation of its target transcript SUPPRESSOR OF MAX2 1-LIKE4/5 (SMXL4/5) (Cho et al., 2018). This study highlights the strong impact of RBPs affecting RG4 folding, other RBPs in plants affecting RG4 folding need to be identified.

Whether RG4 can fold *in vivo* has been a long-standing question. Quantitative measurement of the folding status reveals low folding scores close to 0 in mammalian cells and yeast cells, suggesting RG4 is generally unfolded (Guo and Bartel, 2016). In contrast, the folding scores in both monocot rice and dicot *Arabidopsis* are high, with a median value of 0.9 or 0.7 respectively, suggesting RG4 is folded in plants (Yang et al., 2020). Therefore, RG4 folding status is likely to be different in one species from another. Although the factors that affect RG4 folding in living cells are to be identified, the cellular conditions may be a key factor. Two recent studies have revealed that RG4 folding in both mammalian cells and plants was strongly promoted by stress, such as cold stress (Kharel et al., 2022; Yang et al., 2022). The growth temperature seems to be a key factor affecting RG4 folding in cells, a lower growth temperature of *Arabidopsis* and rice than that in mammalian cells (22°C in *Arabidopsis* and 28°C in rice vs. 37°C in mouse cells) may have contributed to the stronger folding status of RG4 in plants. Incubation of RG4-specific ligands, such as pyridostatin (PDS) promotes the RG4 folding in mammalian cells (Guo and Bartel, 2016; Weng et al., 2020), further supporting the pivotal impact of cellular conditions in affecting RG4 folding *in vivo*. The key feature of condition-dependent folding of RG4s may have enabled RG4 to function as a general molecular sensor for environments.

## Methods for RNA G-Quadruplex Detection

RG4 structure folding requires specific features of G-rich sequence, and several sequence-based algorithms have been developed to predict putative RG4 structure, which has been well summarized by other publications (Table 1; Puig Lombardi and Londoño-Vallejo, 2020). The first type of tool, such as Quadparser (Huppert and Balasubramanian, 2005), was developed based on regular expression matching for the putative G4 motif, G_x_N_L_G_x_N_L_G_x_N_L_G_x_, where G represents G and N represents A, C, G or U, x denotes the number of G-quartet layers while L denotes the length of loops connecting G-tracts. Using Quadparser, a high prevalence of RG4s was found in both dicots and monocots (Mullen et al., 2010; Garg et al., 2016). The hit of the G4 feature is largely dependent on the artificial settings on the x and L parameters. While the setting on x is generally 2 to 3, the setting on L is much more variable, for example, 1 to 7 nt in Quadparser. Although an increase in loop length can strongly decrease the RG4 stability, RG4 with a longer loop is likely to form, the longest loop up to 15 nt for G3-RG4s and longest loop up to 9 nt for G2-RG4s was applicable with experimental validation when detecting RG4 folding *in vitro* (Kwok et al., 2016a; Yang et al., 2020). The second type of tools, such as QGRS Mapper and G4Hunter predict putative G4s using a scoring system based on the features that stabilize or destabilize G4 formation (Kikin et al., 2006; Bedrat et al., 2016). QGRS mapper mainly concerns the number of G-quartet layers and the loop lengths: A G-rich sequence with more G-quartet layers and shorter loop lengths is likely to generate a high G4-score, suggesting a higher possibility of G4 formation (Kikin et al., 2006). G4Hunter takes into account G-richness and G-skewness represented by G4Hscore, the G4Hscores for A, U, C, and G were denoted as 0, 0,−1, and 1, respectively (Bedrat et al., 2016). Therefore, the presence of G or C could strongly increase or decrease the G4Hscore of a given sequence. Higher G4Hscore suggests a higher possibility of G4 formation while a lower G4Hscore suggests a lower possibility of G4 formation. Since G4Hunter scores C as negative, a richness on C is likely to decrease the G4Hscore and proposes the folding of canonical RNA structure such as stem-loop through G-C base-pairing. Therefore, a competition effect between canonical RNA structure and G4 structure is potentially embedded in G4Hunter theoretically. Regarding the competition between G4 and alternative RNA secondary structures, a comparison of the folding energy of different structures was applied to estimate the folding possibility of RG4 in Vienna RNAfold (Lorenz et al., 2013).

**Table 1:** Methods to study RG4.

**Method**	**Characterization on RG4**	**References**
NMR	3D structure *in vitro*	Smith and Feigon, 1992
CD	Topology *in vitro*	Vorlíčková et al., 2012
UV-thermal melting	Thermostability *in vitro*	Mergny et al., 1998
QGRS mapper	Prediction *in silico*	Kikin et al., 2006
Quadparser	Prediction *in silico*	Huppert and Balasubramanian, 2005
Intrinsic fluorescence	Detection *in vitro*	Huang et al., 2014
BG4	Visualization in fixed cells	Biffi et al., 2014
G4Hunter	Prediction *in silico*	Bedrat et al., 2016
rG4-seq	Single motif *in vitro*	Kwok et al., 2016a
DMS based seq	Quantitative measurement at single motif *in vivo*	Guo and Bartel, 2016
QUMA-1	Visualization in living cells	Chen et al., 2018
SHALiPE-seq	Quantitative measurement at single motif *in vivo*	Yang et al., 2020
Keth-seq	Detection at single motif *in vivo*	Weng et al., 2020

*Summation of the key methods for RNA G-quadruplex prediction and detection. The method names, their application, and references are listed*.

*In vitro* folding of RG4 can be detected using biophysical methods (Table 1). Circular dichroism (CD) spectra characterize parallel RG4 folding with a positive peak near 260 nm while a negative peak near 245 nm (Vorlíčková et al., 2012). While using UV-thermal melting analysis, the characteristic peak associated with the G4 structure appears at 295 nm (Mergny et al., 1998). Besides, intrinsic fluorescence of nucleic acids with a peak around 390 nm represents the formation of RG4 *in vitro* (Kwok et al., 2013). Nuclear magnetic resonance (NMR) spectroscopy is widely used, which allows the determination of RG4 structure at atomic resolution (Smith and Feigon, 1992; Webba da Silva, 2007). The methods mentioned above are commonly used to validate RG4 formation in a complementary way, and can only determine RG4 with low throughput.

High-throughput methods rG4-seq and profiling of reverse transcription (RT) stalling determine RG4 formation *in vitro* at transcriptome-wide (Table 1; Balasubramanian and Neidle, 2009; Kwok et al., 2016a; Yang et al., 2020). rG4-seq was developed based on the two facts: (1) RG4 formation is strongly dependent on the cation, Li^+^ destabilizes while K^+^ stabilizes RG4, ligands such as PDS preferably stabilize RG4 in the presence of K^+^ (Bugaut et al., 2010); and (2) RG4 folding with high stability can cause RT stalling (Kwok and Balasubramanian, 2015). Therefore, RT stalling dependent on K^+^ or K^+^+PDS represents RG4 formation *in vitro*. rG4-seq enabled the identification of thousands of RG4 structures in human and *Arabidopsis* transcriptomes (Kwok et al., 2016a; Yang et al., 2020). Notably, the vast majority of *Arabidopsis* G2-RG4s detected in the presence of K^+^+PDS were not identified when in the presence of only K^+^, especially for the G2-RG4s with long loops (Yang et al., 2020). This could be explained by the relatively low stability of G2-RG4, which is not strong enough to cause RT stalling. As such, in addition to the detection of RT stalling by K^+^, detection of RT stalling by K^+^+PDS is strongly recommended when performing rG4-seq to generate a full map of RG4 structures.

RG4-specific antibodies and fluorescent probes allowed the visualization of RG4 in cells (Table 1; Biffi et al., 2014; Huang et al., 2014). Through a phage display with a library of over 10^10^ antibody clones, an antibody showing high affinity to G4 structures was selected. This antibody, termed BG4, was broadly used to visualize both DNA G-quadruplex and RNA G-quadruplex in cells (Biffi et al., 2013, 2014). Since BG4-based visualization can only detect RG4 structure in fixed cells, an effort was paid to develop the methods for visualization of RG4 in living cells. The RG4-specific fluorescent probe, QUMA-1, which can penetrate the cells, allows the visualization of RG4 in a selective, continuous, and real-time way (Chen et al., 2018). These methods are particularly powerful for RG4 visualization at the cellular level, and competent for quantitatively measuring RG4 intensity in single cells. Concerns have been raised on the robustness of these methods in terms of side effects, such as triggering RG4 formation in cells. Given these methods have been originally developed in mammalian cells, necessary modification should be evaluated when applying these methods to plants as plant cell walls may cause additional difficulties for these ligands penetrating the cells.

To evaluate RG4 folding at a single transcript, a method combining G4-RNA-specific precipitation (G4RP) and sequencing was developed, termed G4RP-seq (Yang S. Y. et al., 2018). After crosslinking by formaldehyde, transcripts with RG4 folding were enriched by a G4-specific ligand BioTASQ and subjected to sequencing. G4RP-seq can quantify the enrichment index on an individual transcript, which represents the RG4 folding strength on the mRNAs. One major drawback for G4RP-seq is that ligand-binding affinity on individual G4 may be different, therefore causing uncertainties in the enrichment analysis. Other uncertainties could be also brought by the side effect of crosslinking with formaldehyde, which is likely to cause RNA degradation. Therefore, optimization of experimental procedures may be required when applying G4RP-seq in plants.

To quantitatively measure the folding status of individual RG4-motif, three chemical profiling methods have been developed, based on the small molecules dimethyl sulfate (DMS) (Guo and Bartel, 2016), 1,1-dihydroxy-3-ethoxy-2-butanone (kethoxal) (Weng et al., 2020), and 2-methylnicotinic acid imidazolide (NAI) (Kwok et al., 2016b), respectively. The DMS method was developed based on its modification of the N7G when RG4 is unfolded. Therefore, if RG4 is folded *in vivo*, the N7G position (marked by number 7 in orange in Figure 1A) is protected against modification under high DMS concentration and RNA can fold into RG4 structure *in vitro* again in the presence of K^+^ when performing reverse transcription, and subsequently cause strong RT stalling. However, the DMS-based method is unlikely to work with high performance in plants due to two reasons: (1) high concentration of DMS to promise an over-modification will cause significant browning of plants and RNA degradation (Wang et al., 2019); (2) most *Arabidopsis* G2-RG4s are unlikely to fold into structures stable enough to cause strong RT stalling at a temperature over 37°C, as such, these RG4s may be always “unfolded” in detection no matter if they fold or not *in vivo*. Kethoxal modifies the N1G and N2G positions, therefore, when RG4 is folded, the N1G and N2G positions (marked by numbers 1 and 2, respectively in orange in Figure 1A) are likely to be stronger modified and cause RT stalling (keth-seq). Notably, while the DMS-based method quantifies RG4 generally unfolded in mouse embryonic stem cells (mESCs) (Guo and Bartel, 2016), keth-seq argues a small proportion of RG4s can fold *in situ* in mESCs (Weng et al., 2020). Due to the recent development of SHAPE-seq, there have been published studies showing the performance of SHAPE-seq in RG4 detection in plants (Kwok et al., 2016b; Yang et al., 2020). The SHAPE reagent NAI shows specific modification on folded RG4s, that strongly acylates the 2-OH of ribose on the last G of G-tracts rather than the first G of G-tracts. This NAI modification can further result in strong RT stalling in the presence of Li^+^, termed as SHALiPE-seq. Reverse transcription with Li^+^ won't trigger RG4 folding and additional RT stalling, therefore it can robustly detect the signal caused by NAI modification *in vitro* or *in vivo*. SHALiPE-seq quantitatively measured the RG4 folding status in plants and detected hundreds of RG4s folded in both *Arabidopsis* and rice (Yang et al., 2020). Since SHALiPE-seq doesn't require a refolding of RG4 *in vitro* when performing reverse transcription, it can measure the folding status of different types of RG4s regardless of their stability. Moreover, SHALiPE-seq probes RNA structure under a relatively low concentration without over-modification, it won't cause significant harm to plants. Therefore, SHALiPE-seq is likely to provide the most robust result of RG4 detection in plants up to date.

## The Molecular Functions of RG4 in Gene Regulation

Due to its high stability, RG4 has been suggested to play key roles in gene regulation for a long time. The most prevalent regulatory role suggested is translational inhibition, in both mammalian cells and plants (Frees et al., 2014; Dumas et al., 2021). A lower translation efficiency on transcripts with RG4s than that without RG4s at the transcriptome level reveals a general effect of RG4 in repressing translation (Yang et al., 2020). The first complete demonstration of plant RG4 was shown within the 5′-UTR of the ATAXIA TELANGIECTASIA-MUTATED AND RAD3-RELATED (ATR), and the formation of G3 type RG4 strongly represses ATR translation in *Arabidopsis* (Figure 1B) (Kwok et al., 2015). This RG4 is conserved in plants, suggesting a similar role of this RG4 in other plants. A link between RG4 and plant physiological significance was revealed by RG4-mediated translational control in phloem development (Cho et al., 2018). An RNA binding protein (RBP) JULGI can promote RG4 formation *in vitro*, and subsequently represses translation of downstream genes, such as SUPPRESSOR OF MAX2 1-LIKE4/5 (SMXL4/5) (Cho et al., 2018). Knock-down of JULGI or SMXL4/5 caused significant changes in phloem development compared to wild-type plants in both *Arabidopsis* and tobacco. Although this study lacks the *in vivo* proof of RG4 formation, it highlights the complex regulation of RG4 formation and its functionality in gene regulation and phenotypic contribution. Disruption of the *in vivo* folded RG4 on Hird11 resulted in a higher translation of HIRD11 and longer root length in *Arabidopsis*, showing the first proof of phenotypic effect by a single RG4 structure *in vivo* in eukaryotes (Yang et al., 2020).

Besides translational control, other molecular functions of RG4 such as alternative polyadenylation (Beaudoin and Perreault, 2013), telomere maintenance (Takahama et al., 2013) and miRNA binding (Stefanovic et al., 2015) have been revealed. In human cells, RG4 is suggested to promote exon inclusion, therefore plays a role in regulating alternative splicing (Huang et al., 2017; Weldon et al., 2018). Although there is still a lack of knowledge about whether RG4 may regulate mRNA processing in plants, it's of great interest to explore.

The RG4 structure, but not a mutant RG4 in the CDS of the SHORT ROOT (SHR) mRNA triggers the liquid-liquid phase separation of SHR mRNA (Figure 1B) (Zhang et al., 2019). RG4 mediated phase separation results in the formation of membrane-less granules which may have greatly contributed to the movement of SHR mRNA. Notably, the RG4-mediated phase separation is influenced by the intrinsic features of RG4s, such as the number of G-quartets and the length of loops, more G-quartets and longer loops provide a better trigger for RNA phase separation. Since thousands of mRNAs are mobile (Thieme et al., 2015), different intrinsic features of RG4s may have been adopted to contribute to mRNA movement.

The novel function of RG4 in regulating mRNA decay has been revealed recently. Plant RG4s in 3'UTRs are especially sensitive to cold temperature and are associated with the higher stability of mRNAs (Figure 1B) (Yang et al., 2022). Disruption of RG4s significantly decreased mRNA stability in cold, therefore affecting plant phenotypic response to cold temperature. This study, along with the other study in human cells (Kharel et al., 2022), highlights the new molecular functions of RG4s that were largely unacknowledged before in eukaryotes.

The genetic regions competent to fold into RNA are satisfied with the sequence requirement of folding into DNA G4 structures. The vast majority of studies have shown that while RG4 structures mostly regulate gene expression at post-transcriptional levels, DNA G4 structures play key roles in genome instability, telomere biology, and transcriptional regulation, a comprehensive comparison between the functionality of RG4 structures and that of DNA G4 structures is summarized by the other literature (Varshney et al., 2020). Notably, emerging evidence has shown that the functionality of RG4 at the transcriptional level, for example, RG4 structure can be converted to DNA:RNA hybrid R-loop (de Almeida et al., 2018; Caterino and Paeschke, 2021), emphasizing G4 structure may have enabled coupling regulation between DNA and RNA.

## Conclusion and Perspective

Although it's been decades since the characterization of G4 structure by nucleic acids chemistry, the knowledge of RNA G-quadruplex has been significantly lagging behind the DNA G-quadruplex. Different tools predicting RG4 have significantly promoted the discovery of RG4s in kinds of species, yet the prediction still suffers from considerable false-positive rates and false-negative rates. Sensitive and effective methods of detecting RG4 *in vivo* are to be developed to advance our knowledge of RG4 *in vivo*, which may be applied to improve the predictive power of RG4 folding in living organisms (Chen et al., 2018; Yang S. Y. et al., 2018; Yang et al., 2020). The dynamics of RG4 folding and the factors affecting RG4 folding are of particular interest, which may tightly link with plant physiology. For example, the cations such as K^+^, and NH4^+^ that can stabilize RG4 formation may be adopted by plants to control RG4 formation *in vivo* to regulate plant nutrient utilization. Given the strong impact of temperature on RG4 folding, RG4 may have been also widely adopted for plant sensing a broad range of temperature fluctuations. Selection of the key RG4s on specific genes, within specific regions, and in specific plants may have strongly contributed to plant evolution and adaptation. Key RG4s regulating plant development and environmental adaptation may be applied in future breeding for crop improvement.

## Author Contributions

All authors listed have made a substantial, direct, and intellectual contribution to the work and approved it for publication.

## Funding

HL received an academic visitor scholarship (202009135001) from the China Scholarship Council. HL and ZC are supported by the National Natural Science Foundation of China (31801722) and the Shandong Modern Agricultural Technology & Industry system (SDAIT-17-06). XY is supported by the starting grant of the Chinese Academy of Sciences and John Innes Centre.

## Conflict of Interest

The authors declare that the research was conducted in the absence of any commercial or financial relationships that could be construed as a potential conflict of interest.

## Publisher's Note

All claims expressed in this article are solely those of the authors and do not necessarily represent those of their affiliated organizations, or those of the publisher, the editors and the reviewers. Any product that may be evaluated in this article, or claim that may be made by its manufacturer, is not guaranteed or endorsed by the publisher.

## References

[B1] Balasubramanian S. Neidle S. (2009). G-quadruplex nucleic acids as therapeutic targets. Curr. Opin. Chem. Biol. 13, 345–353. 10.1016/j.cbpa.2009.04.637 19515602 PMC2726962

[B2] Beaudoin J. D. Jodoin R. Perreault J. P. (2013). New scoring system to identify RNA G-quadruplex folding. Nucleic Acids Res. 42, 1209–1223. 10.1093/nar/gkt904 24121682 PMC3902908

[B3] Beaudoin J. D. Perreault J. P. (2013). Exploring mRNA 3′-UTR G-quadruplexes: evidence of roles in both alternative polyadenylation and mRNA shortening. Nucleic Acids Res. 41, 5898–5911. 10.1093/nar/gkt265 23609544 PMC3675481

[B4] Bedrat A. Lacroix L. Mergny J. L. (2016). Re-evaluation of G-quadruplex propensity with G4Hunter. Nucleic Acids Res. 44, 1746–1759. 10.1093/nar/gkw006 26792894 PMC4770238

[B5] Biffi G. Di Antonio M. Tannahill D. Balasubramanian S. (2014). Visualization and selective chemical targeting of RNA G-quadruplex structures in the cytoplasm of human cells. Nat. Chem. 6, 75–80. 10.1038/nchem.1805 24345950 PMC4081541

[B6] Biffi G. Tannahill D. McCafferty J. Balasubramanian S. (2013). Quantitative visualization of DNA G-quadruplex structures in human cells. Nat. Chem. 5, 182–186. 10.1038/nchem.1548 23422559 PMC3622242

[B7] Bugaut A. Rodriguez R. Kumari S. Hsu S. T. D. Balasubramanian S. (2010). Small molecule-mediated inhibition of translation by targeting a native RNA G-quadruplex. Org. Biomol. Chem. 8, 2771–2776. 10.1039/c002418j 20436976 PMC3074098

[B8] Caterino M. Paeschke K. (2021). Action and function of helicases on RNA G-quadruplexes. Methods. 9, 3. 10.1016/j.ymeth.2021.09.003 34509630 PMC9236196

[B9] Chen X. C. Chen S. B. Dai J. Yuan J. H. Ou T. M. Huang Z. S.. (2018). Tracking the dynamic folding and unfolding of RNA G-quadruplexes in live cells. Angew. Chem. Int. Ed. Engl. 130, 4792–4796. 10.1002/ange.201801999 29453903

[B10] Cho H. Cho H. S. Nam H. Jo H. Yoon J. Park C.. (2018). Translational control of phloem development by RNA G-quadruplex–JULGI determines plant sink strength. Nat. Plants 4, 376–390. 10.1038/s41477-018-0157-2 29808026

[B11] de Almeida C. R. Dhir S. Dhir A. Moghaddam A. E. Sattentau Q. Meinhart A.. (2018). RNA helicase DDX1 converts RNA G-quadruplex structures into R-loops to promote IgH class switch recombination. Mol. Cell 70, 650–662. 10.1016/j.molcel.2018.04.001 29731414 PMC5971202

[B12] Dumas L. Herviou P. Dassi E. Cammas A. Millevoi S. (2021). G-Quadruplexes in RNA biology: recent advances and future directions. Trends Biochem. Sci. 46, 270–283. 10.1016/j.tibs.2020.11.001 33303320

[B13] Frees S. Menendez C. Crum M. Bagga P. S. (2014). QGRS-Conserve: a computational method for discovering evolutionarily conserved G-quadruplex motifs. Hum. Genomics 8, 1–13. 10.1186/1479-7364-8-8 24885782 PMC4017754

[B14] Garg R. Aggarwal J. Thakkar B. (2016). Genome-wide discovery of G-quadruplex forming sequences and their functional relevance in plants. Sci. Rep. 6, 1–13. 10.1038/srep28211 27324275 PMC4914980

[B15] Guo J. U. Bartel D. P. (2016). RNA G-quadruplexes are globally unfolded in eukaryotic cells and depleted in bacteria. Science 353, aaf5371. 10.1126/science.aaf5371 27708011 PMC5367264

[B16] Huang H. Suslov N. B. Li N. S. Shelke S. A. Evans M. E. Koldobskaya Y.. (2014). A G-quadruplex–containing RNA activates fluorescence in a GFP-like fluorophore. Nat. Chem. Biol. 10, 686–691. 10.1038/nchembio.1561 24952597 PMC4104137

[B17] Huang H. Zhang J. Harvey S. E. Hu X. Cheng C. (2017). RNA G-quadruplex secondary structure promotes alternative splicing *via* the RNA-binding protein hnRNPF. Genes Dev. 31, 2296–2309. 10.1101/gad.305862.117 29269483 PMC5769772

[B18] Huppert J. L. Balasubramanian S. (2005). Prevalence of quadruplexes in the human genome. Nucleic Acids Res. 33, 2908–2916. 10.1093/nar/gki609 15914667 PMC1140081

[B19] Jana J. Weisz K. (2021). Thermodynamic Stability of G-quadruplexes: impact of sequence and environment. ChemBioChem. 22, 2848–2856. 10.1002/cbic.202100127 33844423 PMC8518667

[B20] Kharel P. Becker G. Tsvetkov V. Ivanov P. (2020). Properties and biological impact of RNA G-quadruplexes: from order to turmoil and back. Nucleic Acids Res. 48, 12534–12555. 10.1093/nar/gkaa1126 33264409 PMC7736831

[B21] Kharel P. Fay M. Manasova E. V. Anderson P. J. Kurkin A. V. Guo J. U.. (2022). Stress promotes RNA G-quadruplex folding in human cells. bioRxiv. 10.1101/2022.03.03.482884 PMC9839774 36639366

[B22] Kikin O. D'Antonio L. Bagga P. S. (2006). QGRS Mapper: a web-based server for predicting G-quadruplexes in nucleotide sequences. Nucleic Acids Res. 34, W676–W682. 10.1093/nar/gkl253 16845096 PMC1538864

[B23] Klaff P. Riesner D. Steger G. (1996). RNA structure and the regulation of gene expression. Plant Mol. Biol. 32, 89–106. 10.1007/978-94-009-0353-1_5 8980476

[B24] Kwok C. K. Balasubramanian S. (2015). Targeted detection of G-quadruplexes in cellular RNAs. Angew. Chem. Int. Ed. 54, 6751–6754. 10.1002/anie.201500891 25907625 PMC4510783

[B25] Kwok C. K. Ding Y. Shahid S. Assmann S. M. Bevilacqua P. C. (2015). A stable RNA G-quadruplex within the 5′-UTR of Arabidopsis thaliana ATR mRNA inhibits translation. Biochem. J. 467, 91–102. 10.1042/BJ20141063 25793418

[B26] Kwok C. K. Marsico G. Sahakyan A. B. Chambers V. S. Balasubramanian S. (2016a). rG4-seq reveals widespread formation of G-quadruplex structures in the human transcriptome. Nat. Methods 13, 841–844. 10.1038/nmeth.3965 27571552

[B27] Kwok C. K. Merrick C. J. (2017). G-quadruplexes: prediction, characterization, and biological application. Trends Biotechnol. 35, 997–1013. 10.1016/j.tibtech.2017.06.012 28755976

[B28] Kwok C. K. Sahakyan A. B. Balasubramanian S. (2016b). Structural analysis using SHALiPE to reveal RNA G-quadruplex formation in human precursor microRNA. Angew. Chem. 128, 9104–9107. 10.1002/ange.201603562 27355429 PMC6680278

[B29] Kwok C. K. Sherlock M. E. Bevilacqua P. C. (2013). Effect of loop sequence and loop length on the intrinsic fluorescence of G-quadruplexes. Biochemistry 52, 3019–3021. 10.1021/bi400139e 23621657

[B30] Lorenz R. Bernhart S. H. Qin J. zu Siederdissen C. H. Tanzer A. Amman F.. (2013). 2D meets 4G: G-quadruplexes in RNA secondary structure prediction. IEEE/ACM Trans. Comput. Biol. Bioinform. 10, 832–844. 10.1109/TCBB.2013.7 24334379

[B31] Mergny J. L. Phan A. T. Lacroix L. (1998). Following G-quartet formation by UV-spectroscopy. FEBS LETT. 435, 74–78. 10.1016/S0014-5793(98)01043-6 9755862

[B32] Morris K. V. Mattick J. S. (2014). The rise of regulatory RNA. Nat. Rev. Genet. 15, 423–437. 10.1038/nrg3722 24776770 PMC4314111

[B33] Mullen M. A. Olson K. J. Dallaire P. Major F. Assmann S. M. Bevilacqua P. C. (2010). RNA G-Quadruplexes in the model plant species Arabidopsis thaliana: prevalence and possible functional roles. Nucleic Acids Res. 38, 8149–8163. 10.1093/nar/gkq804 20860998 PMC3001093

[B34] Pandey S. Agarwala P. Maiti S. (2013). Effect of loops and G-quartets on the stability of RNA G-quadruplexes. J. Phys. Chem. B. 117, 6896–6905. 10.1021/jp401739m 23683360

[B35] Puig Lombardi E. Londoño-Vallejo A. (2020). A guide to computational methods for G-quadruplex prediction. Nucleic Acids Res. 48, 1–15. 10.1093/nar/gkz1097 31943112 PMC7026631

[B36] Smith F. W. Feigon J. (1992). Quadruplex structure of Oxytricha telomeric DNA oligonucleotides. Nature 356, 164–168. 10.1038/356164a0 1545871

[B37] Stefanovic S. Bassell G. J. Mihailescu M. R. (2015). G quadruplex RNA structures in PSD-95 mRNA: potential regulators of miR-125a seed binding site accessibility. Rna 21, 48–60. 10.1261/rna.046722.114 25406362 PMC4274637

[B38] Takahama K. Takada A. Tada S. Shimizu M. Sayama K. Kurokawa R.. (2013). Regulation of telomere length by g-quadruplex telomere DNA- and TERRA-binding protein TLS/FUS. Chem. Biol. 20, 341–350. 10.1016/j.chembiol.2013.02.013 23521792

[B39] Taylor K. Sobczak K. (2020). Intrinsic regulatory role of RNA structural arrangement in alternative splicing control. Int. J. Mol. Sci. 21, 5161. 10.3390/ijms21145161 32708277 PMC7404189

[B40] Thieme C. J. Rojas-Triana M. Stecyk E. Schudoma C. Zhang W. Yang L.. (2015). Endogenous Arabidopsis messenger RNAs transported to distant tissues. Nat. Plants 1, 1–9. 10.1038/nplants.2015.25 27247031

[B41] Varshney D. Spiegel J. Zyner K. Tannahill D. Balasubramanian S. (2020). The regulation and functions of DNA and RNA G-quadruplexes. Nat. Rev. Mol. Cell Biol. 21, 459–474. 10.1038/s41580-020-0236-x 32313204 PMC7115845

[B42] Vorlíčková M. Kejnovská I. Sagi J. Renčiuk D. Bednárová K. Motlová J.. (2012). Circular dichroism and guanine quadruplexes. Methods 57, 64–75. 10.1016/j.ymeth.2012.03.011 22450044

[B43] Wan Y. Kertesz M. Spitale R. C. Segal E. Chang H. Y. (2011). Understanding the transcriptome through RNA structure. Nat. Rev. Genet. 12, 641–655. 10.1038/nrg3049 21850044 PMC3858389

[B44] Wang Z. Wang M. Wang T. Zhang Y. Zhang X. (2019). Genome-wide probing RNA structure with the modified DMS-MaPseq in Arabidopsis. Methods 155, 30–40. 10.1016/j.ymeth.2018.11.018 30503825

[B45] Webba da Silva M. W. (2007). NMR methods for studying quadruplex nucleic acids. Methods 43, 264–277. 10.1016/j.ymeth.2007.05.007 17967697

[B46] Weldon C. Dacanay J. G. Gokhale V. Boddupally P. V. L. Behm-Ansmant I. Burley G. A.. (2018). Specific G-quadruplex ligands modulate the alternative splicing of Bcl-X. Nucleic Acids Res. 46, 886–896. 10.1093/nar/gkx1122 29156002 PMC5778605

[B47] Weng X. Gong J. Chen Y. Wu T. Wang F. Yang S.. (2020). Keth-seq for transcriptome-wide RNA structure mapping. Nat. Chem. Biol. 16, 489–492. 10.1038/s41589-019-0459-3 32015521 PMC7182492

[B48] Yang S. Y. Lejault P. Chevrier S. Boidot R. Robertson A. G. Wong J. M.. (2018). Transcriptome-wide identification of transient RNA G-quadruplexes in human cells. Nat. Commun. 9, 1–11. 10.1038/s41467-018-07224-8 30413703 PMC6226477

[B49] Yang X. Cheema J. Zhang Y. Deng H. Duncan S. Umar M. I.. (2020). RNA G-quadruplex structures exist and function in vivo in plants. Genome Biol. 21, 1–23. 10.1186/s13059-020-02142-9 32873317 PMC7466424

[B50] Yang X. Yang M. Deng H. Ding Y. (2018). New era of studying RNA secondary structure and its influence on gene regulation in plants. Front. Plant Sci. 9, 671. 10.3389/fpls.2018.00671 29872445 PMC5972288

[B51] Yang X. Yu H. Duncan S. Zhang Y. Cheema J. Miller J. B.. (2022). RNA G-quadruplex structure contributes to cold adaptation in plants. bioRxiv. 10.1101/2022.03.04.482910 PMC9585020 36266343

[B52] Zhang Y. Yang M. Duncan S. Yang X. Abdelhamid M. A. Huang L.. (2019). G-quadruplex structures trigger RNA phase separation. Nucleic Acids Res. 47, 11746–11754. 10.1093/nar/gkz978 31722410 PMC7145655

